# Erratum, Vol. 1, No. 1

**Published:** 2004-03-15

In the book review of *Community-Based Health Research: Issues and Methods*, one of the editors, Daniel S. Blumenthal, was incorrectly identified as David S. Blumenthal. The information was corrected on our Web site on Monday, December 15, 2003, and the correct information appears below:


Community-Based Health Research: Issues and Methods

Editors: Daniel S. Blumenthal, MD, MPH, Ralph J. DiClemente, PhD
New York
Springer Publishing Company
Publication Date: 27 Oct 2003
240 pages
Price: $39.95 (domestic), $44.80 (foreign)
ISBN: 0-8261-2025-3

When a book on community-based research opens with comments about Imhotep, Hippocrates, and Aesculapius, it certainly catches the reader's attention. Such is the case with *Community-Based Health Research: Issues and Methods*, a new book edited by Daniel S. Blumenthal and Ralph J. DiClemente.

The mistake appeared in the introductory information for the book as well as in the first paragraph of the review. The corrected article also appears online at http://www.cdc.gov/pcd/issues/2004/jan/03_0031.htm.

We regret any confusion this error may have caused.

